# Notes and News

**Published:** 1897-01-23

**Authors:** 


### NOTES AND NEWS.

(COLLECTED AND COMMUNICATED.)

THE new fever hospital for East Stirlingshire contains forty beds, and was erected at a cost of £7,500.

LORD ROTHSCHILD has accepted the office of treasurer of the West London Hospital.

A DINNER in aid of the funds of the Association for the Oral Instruction of the Deaf and Dumb will be held at the Whitehall Rooms, Hotel Metropole, on March 17th, at which the Duke of York will preside.

IT is reported that Dr. Niels Finsen has established a Blue Light Hospital in Copenhagen. Dr. Finsen's method of condensing the blue rays is utilised for treatment, and in skin diseases the patient wears a glass shape, which is described as pressing the blood away from the affected part, as he is reported to find that the blood does not allow of the passage of actinic rays. Dr. Finsen has constructed a condenser to be used in connection with the electric light, which in some circumstances is found to be much quicker in action than the sun rays.

AT the annual meeting of the Edinburgh Royal Infirmary, Dr. Batty Tuke brought forward his objection to the proposed site for the new laundry in connection with the institution. He said that whilst the site was a bad one for a laundry owing to its enclosed position, it also would cause the occupation of the only ground available for future extension of the infirmary. He suggested that the laundry should be established in the country, and that instead of a laundry directly connected with the institution a much needed out-patients' department should be erected. During the discussion which followed it was intimated that the estimates for the erection of the laundry on the site objected to by Dr. Tuke had been actually accepted, and some present contended that the work was actually begun. The discussion ended by the resolution to elect a Committee of Contributors to report on the matter.

THE Duke of Fife has consented to preside at a dinner in aid of the Alexandra Hospital for Children with Hip Disease on Thursday, April 8th, at the Hotel Metropole.

THE news from Bombay continues to be most depressing. It is almost impossible to enforce proper sanitary measures amongst the native population, who are at the same time panic-sticken. Since the outbreak of the plague 2,708 deaths have been reported. The European countries with seaports are taking precautions to prevent the introduction of the disease, and a general feeling prevails that very great precautions should be taken in respect to the Mecca pilgrimages, as likely to be grave sources of danger.

The Kilmarnock Infirmary is to be especially congratulated, for we learn from its annual report that the workpeople's contributions exceed the annual subscriptions of the well-to-do. There is no question that provincial hospitals have the advantage over metropolitan institutions, inasmuch as they secure far more local interest, and this interest again is most apparent at Kilmarnock. The annual report contains a list of contributions in kind from the neighbourhood, and a perusal of the list is quite an education in giving. All who have had a little to spare in one form or another seem to have thought of the infirmary, and thus the kindly interest is kept alive, and the ball of kindly feeling grows larger to the benefit not only of the recipients, the needy poor, but of the whole community, knit together by common interests.

THE Hospital Saturday Fund is making the following appeal:—

"May we appeal through you to the public for assistance in purchasing and fitting up suitable premises for the Hospital Saturday Fund? The large amount of work which is done by the fund, including the supplying of surgical appliances on a carefully thought-out system of part payment by instalments, of instructing workpeople in first aid, in connection with the St. John Ambulance Association, and thus fitting them to render immediate assistance in cases of accident in the workshops and in other directions, besides the important work of collecting money for the hospitals, dispensaries, and convalescent homes, has made it absolutely necessary to leave the present inconvenient premises in Farringdon Road. An opportunity offers of purchasing the freehold of suitable premises in a central position, easy of access from all parts of London, for the sum of £4,000, and, by the expenditure of an additional sum of about £500, these premises can be thoroughly adapted to the increased work of the fund. If this sum can be raised the present charge of £180 a year for rent will be extinguished, and the fund would have a permanent home in which to carry on its most useful work. The total collected last year by the fund exceeded £20,000, of which over £15,000 came from workshops and places of business in London, showing a very satisfactory increase. We, therefore, confidently appeal for help in order to provide this association with a permanent centre for its work. Donations may be sent to the treasurer, or to the secretary, Mr. W. G. Bunn, at the offices of the fund, 59, Farringdon Road, E.C.—Your obedient servants,

"G. FAUDEL-PHILLIPS, Lord Mayor, President.
"R. B. D. ACLAND, Chairman.
"H. N. HAMILTON HOARE, Treasurer.
"59, Farringdon Road, E.C., January 15th."

